# Transformational coaching and technical-tactical performance efficiency ratio in basketball: an empirical analysis

**DOI:** 10.3389/fpsyg.2026.1884143

**Published:** 2026-08-07

**Authors:** Xiaofen Cao, Wan Ahmad Munsif Wan Pa

**Affiliations:** Faculty of Education, Universiti Kebangsaan Malaysia, Bangi, Selangor, Malaysia

**Keywords:** athletes' technical-tactical performance, technical-tactical performance efficiency ratio, transformational basketball coach, transformational leadership theory, youth basketball athletes

## Abstract

This study aims to explore the impact of transformational basketball coaching on athletes' technical-tactical performance and its efficiency ratio. A quantitative research design integrating questionnaire surveys and controlled experimental comparisons was adopted. A total of 306 basketball athletes and their coaches were recruited as the initial sample, with 286 valid samples ultimately retained for data analysis. The study developed and validated evaluation scales and calculation tools for core variables, and implemented a 6-month longitudinal tracking with pre-test, mid-test and post-test measurements for both the experimental group and the control group. Post-test results indicate that the experimental group under transformational coaching achieved significant improvements: the comprehensive technical score reached 7.15, the tactical execution rate reached 34.67%, and the technical-tactical performance efficiency ratio reached 7.02. All these indicators were significantly higher than those of the control group, with *t*-values of 8.26, 10.53 and 12.38 respectively and all *p*-values below 0.01.

## 1 Introduction

Basketball has developed into one of the most competitive and tactically demanding team sports, requiring athletes to integrate technical proficiency, tactical awareness, decision-making ability, and teamwork under rapidly changing game conditions. As the complexity of modern basketball continues to increase, the role of coaches has gradually shifted from simply transmitting technical knowledge to facilitating athletes' comprehensive development through effective leadership. Previous studies have demonstrated that coaching methods and leadership styles play an essential role in promoting athletes' positive development, competitive behavior, and long-term performance improvement (De Albuquerque et al., 2021). Among various leadership approaches, transformational leadership has attracted considerable attention because it enhances team performance by inspiring athletes' intrinsic motivation, strengthening commitment, and fostering collective goals (Mach et al., 2022). Based on transformational leadership theory proposed by Bass and Avolio (1994), coaches influence athletes through four behavioral dimensions, namely idealized influence, inspirational motivation, intellectual stimulation, and individualized consideration. Recent evidence further indicates that athletes who perceive higher levels of transformational leadership from

their coaches report greater subjective wellbeing, stronger motivation, and more positive psychological outcomes, all of which contribute to sustainable athletic development (Liu et al., 2023).

Although transformational leadership has been widely discussed in sport psychology, basketball performance remains fundamentally determined by athletes‘ ability to combine technical execution with tactical implementation during competition. Technical–tactical performance therefore represents one of the most important indicators for evaluating basketball athletes' competitive competence. Existing research has shown that technical and tactical performance differs substantially across playing positions and competition situations, emphasizing the necessity of evaluating both technical skills and tactical execution simultaneously rather than independently (Hatem et al., 2020). Furthermore, a high-quality coach–athlete relationship has been identified as an important predictor of athletes‘ training engagement and skill development (Luo et al., 2025). Effective communication and team cohesion also strengthen the positive relationship between transformational leadership and athletic performance (Oh and Yoo, 2023). In addition, coaching leadership style has been found to influence athlete engagement both directly and indirectly, suggesting that leadership behaviors affect athletes not only psychologically but also behaviorally throughout the training process (Li and Xing, 2025). These findings indicate that transformational coaching may contribute to improving basketball athletes' technical and tactical performance through multiple interconnected mechanisms.

Beyond its influence on athletes, transformational coaching has also become an important component of modern coaching education and leadership development. Studies have emphasized that transformational coaching encourages coaches to apply social and emotional competencies to athlete management and long-term development, thereby creating more supportive training environments (Hebard et al., 2021). Similar positive effects have also been reported in other team sports, where transformational coaching enhances athletes' mental toughness and physical performance, particularly during adolescence when athletes experience rapid technical and psychological development (Murray et al., 2021). Meanwhile, advances in basketball performance evaluation have increasingly incorporated intelligent technologies and scientific analytical methods. Data mining techniques have been introduced to analyze basketball tactics and game performance more accurately (Li, 2025), while tactical consciousness training has been recognized as an effective approach for improving athletes' tactical cognition and decision-making ability during basketball teaching and training (Wang, 2024). Case-based investigations have further demonstrated the importance of practical tactical intelligence in complex offensive situations, particularly in pick-and-roll decision-making (Matsumoto and Aida, 2022). At the same time, quantitative analyses of three-point shooting efficiency have provided new perspectives for evaluating technical effectiveness under different competitive systems (Karamousalidis et al., 2025), and artificial intelligence has gradually been introduced into personalized basketball training programs to maximize athletes' individual development potential through intelligent training design (Li and He, 2025).

Despite these important advances, several limitations remain in the current literature. Existing studies have primarily examined transformational coaching from psychological or behavioral perspectives, whereas relatively few investigations have directly explored its influence on athletes‘ basketball-specific technical–tactical performance using comprehensive quantitative indicators. Moreover, previous studies have usually evaluated technical skills and tactical execution separately, making it difficult to reflect their synergistic contribution to competitive performance. To address these research gaps, the present study integrates transformational leadership theory with basketball technical–tactical performance evaluation and proposes a technical–tactical performance efficiency ratio as a comprehensive indicator of athletes' ability to combine technical execution with tactical implementation. Through a longitudinal experimental design, this study investigates how transformational basketball coaching influences athletes‘ technical performance, tactical execution, and overall technical–tactical efficiency. The findings are expected to enrich the theoretical application of transformational leadership in basketball coaching while providing practical evidence for optimizing coaching strategies and improving athletes' long-term competitive performance.

## 2 Related theoretical foundations and core concept definition

### 2.1 Core concept definition

#### 2.1.1 Transformational basketball coach

Transformational basketball coaching refers to a coaching approach that promotes athletes‘ long-term development by combining technical instruction with psychological guidance, interpersonal support, and leadership behaviors. Unlike traditional coaching models that primarily emphasize skill acquisition and competition outcomes, transformational coaching encourages athletes to actively participate in learning, develop independent thinking, and continuously improve their tactical adaptability. Recent advances in intelligent basketball analysis have further highlighted that effective coaching should facilitate athletes' understanding of tactical structures and dynamic decision-making rather than merely teaching fixed tactical patterns (Lingrui et al., 2025).

Within basketball training, transformational coaching also emphasizes athletes' ability to perceive, recognize, and respond to rapidly changing tactical situations. Studies on tactical identification have shown that improving athletes' perceptual awareness and tactical cognition contributes substantially to decision-making quality during competition, demonstrating that coaching should cultivate both technical competence and tactical intelligence (Naito et al., 2020). From the perspective of coaching professionalism, coaches are expected to possess comprehensive instructional competence, organizational ability, and leadership readiness in order to effectively guide athletes' technical, tactical, and psychological development throughout the training process. Consequently, transformational basketball coaches not only organize training activities but also create an environment that encourages learning, communication, and continuous improvement.

The practical implementation of transformational basketball coaching requires coaches to integrate innovative instructional strategies with individualized athlete development. Research has suggested that optimizing basketball technical and tactical instruction through intelligent algorithms and scientific training methods can significantly improve athletes‘ learning efficiency and tactical execution (Zhen-Feng, 2020). Furthermore, data-driven approaches have demonstrated that performance outcomes are influenced by multiple interacting factors, including technical execution, tactical coordination, injury management, and team organization, indicating that coaching decisions should be supported by comprehensive performance analysis rather than isolated technical evaluation (Sarlis et al., 2021). Therefore, transformational basketball coaching can be regarded as an athlete-centered leadership approach that integrates technical instruction, tactical guidance, scientific decision-making, and individualized support to maximize athletes' long-term competitive development (Kai, 2020).

#### 2.1.2 Athletes' technical-tactical performance

Athletes' technical-tactical performance refers to the integrated ability to execute basketball techniques effectively while applying appropriate tactical decisions during training and competition. Rather than evaluating isolated technical skills or tactical knowledge, this concept emphasizes the coordination between individual technical execution and collective tactical implementation, reflecting athletes' overall competitive competence. Research has demonstrated that transformational leadership can positively influence athletes' objective performance over time by enhancing motivation, behavioral engagement, and the quality of performance execution during competition (Bormann and Rowold, 2015).

Technical-tactical performance consists of two closely related dimensions. Technical performance reflects athletes‘ proficiency in executing fundamental basketball skills, such as dribbling, passing, shooting, rebounding, and defensive movements, whereas tactical performance emphasizes the ability to understand game situations, cooperate with teammates, and implement offensive and defensive strategies effectively. Previous studies have shown that coaches' transformational leadership behaviors are associated with improved athlete outcomes, and this relationship is further influenced by coaches‘ personal characteristics and the educational environment in which athletes develop (Kao et al., 2023). Moreover, athletes' individual values may moderate the effectiveness of coaching leadership on the development of personal skills and team-related competencies, suggesting that technical-tactical performance is shaped by both coaching behaviors and athlete characteristics (Corti et al., 2023).

The evaluation of athletes‘ technical-tactical performance generally combines qualitative assessment with quantitative analysis to provide a comprehensive understanding of competitive ability. Professional evaluations of technical execution are commonly integrated with objective indicators reflecting tactical implementation, allowing coaches to assess athletes' ability to transform individual skills into effective team performance. Because basketball requires continuous interaction among teammates, technical proficiency alone cannot guarantee competitive success without effective tactical coordination. Therefore, technical-tactical performance should be regarded as a multidimensional construct reflecting the combined effects of technical mastery, tactical execution, and team cooperation, all of which contribute to collective efficacy and overall competitive performance (García-Calvo et al., 2014).

#### 2.1.3 Athletes technical-tactical performance efficiency ratio

Athletes technical-tactical performance efficiency ratio is a comprehensive evaluation index that reflects the synergy between athletes‘ technical performance and tactical execution. It is not a simple superposition of single indicators but a ratio that measures the actual effect of athletes' technical-tactical combination based on scientific calculation methods (Luo et al., 2025).

This efficiency ratio takes into account both the comprehensive technical score and the tactical execution rate. It reflects the efficiency of athletes in converting technical skills into tactical results in the process of participating in competitions or training. For example, athletes with high technical scores may not achieve good competitive results if they cannot effectively integrate with team tactics, and their technical-tactical performance efficiency ratio will be relatively low. On the contrary, athletes who can flexibly use technical skills according to tactical requirements will have a higher efficiency ratio.

**Figure 1:**
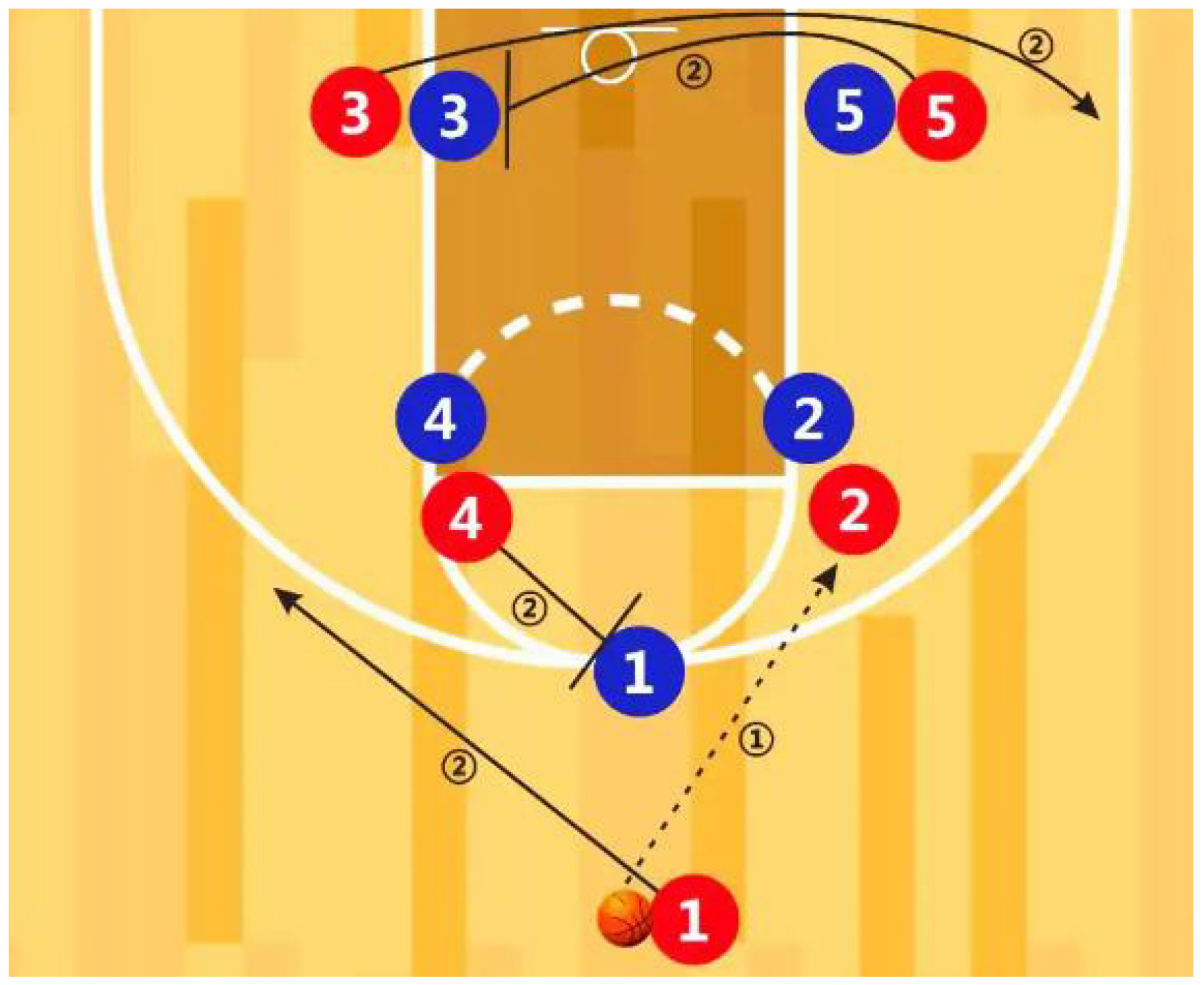
Basketball technical and tactical case: half court man to man tactics.

The construction of the technical-tactical performance efficiency ratio provides a more comprehensive and in-depth evaluation perspective for basketball training and competition. It helps coaches and athletes recognize the weak links between technical mastery and tactical application, and provides a clear direction for targeted training improvement. Compared with single technical or tactical indicators, this efficiency ratio is more in line with the actual needs of basketball competitions that emphasize teamwork and synergy see Figure 1.

### 2.2 Related theoretical foundations

#### 2.2.1 Transformational leadership theory

Transformational leadership theory is an important theory in the field of leadership research, proposed based on the study of organizational management and team development. The core idea of this theory is that leaders can inspire followers' intrinsic motivation and potential through their own behavior and influence, so that followers can surpass their own expectations and achieve the common goals of the team.

This theory divides transformational leadership into four core dimensions: idealized influence, inspirational motivation, intellectual stimulation, and individualized consideration. These dimensions are interrelated and complementary, forming a complete leadership behavior system. In the field of sports, coaches, as the core leaders of the team, their leadership style directly affects the training attitude, skill improvement, and team cohesion of athletes. The application of transformational leadership theory in basketball coaching provides a theoretical framework for defining the behavior characteristics of transformational basketball coaches.

Transformational leadership theory emphasizes the emotional connection and value identification between leaders and followers. For basketball coaches, this means not only focusing on technical and tactical guidance but also paying attention to the psychological needs and value pursuit of athletes. By establishing a good coach-athlete relationship, coaches can enhance athletes' sense of identity and belonging to the team, thereby promoting the overall improvement of the team's competitive level. This theory provides important theoretical support for the design and implementation of this study's research tools and experimental interventions .

#### 2.2.2 Motor performance theory

**Figure 2:**
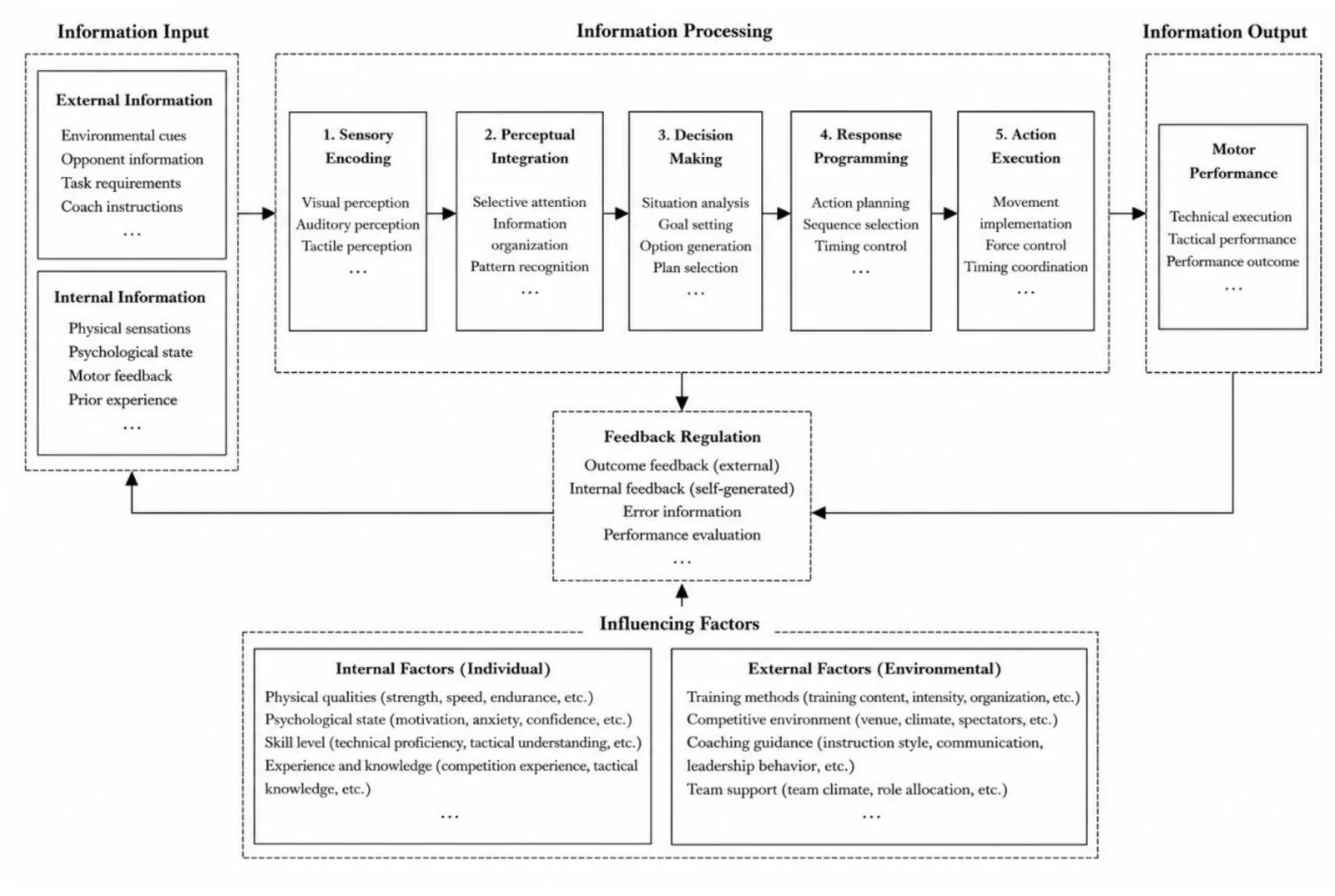
Information processing model.

Motor performance theory is a specialized theory that studies the formation, development, and improvement of human motor behavior. It involves multiple disciplines such as sports science, psychology, and physiology, and focuses on exploring the internal mechanism and external influencing factors of motor performance. The core content of this theory includes the process of motor skill acquisition, the information processing mechanism in motor behavior, and the adaptation of motor performance to environmental changes see Figure 2.

Motor performance theory holds that an individual's motor performance is the result of the interaction of internal factors and external factors. Internal factors include physical condition, psychological state, and skill mastery, while external factors include training methods, competitive environment, and guidance from coaches. For basketball athletes, their technical-tactical performance is a typical motor performance, which is affected by their own physical and psychological conditions, as well as the training methods and leadership styles of coaches.

This theory provides a theoretical basis for the evaluation and improvement of athletes‘ technical-tactical performance. It helps to clarify the key links in the process of athletes' skill acquisition and tactical execution, and provides a scientific basis for the development of research tools such as technical-tactical performance evaluation tools. At the same time, motor performance theory also guides the design of this study's experimental intervention plan, emphasizing that the intervention measures should be consistent with the law of motor performance improvement to ensure the effectiveness of the intervention.

## 3 Research design

### 3.1 Research objects

#### 3.1.1 Scope of sample selection

The research objects of this study consist of basketball athletes and their coaches recruited from different regional youth basketball teams. The athlete participants are aged 15 to 22 years with 2 to 8 years of systematic basketball training experience. This age cohort represents a critical developmental period for motor skill acquisition and tactical cognition, with substantial potential for technical-tactical performance improvement, making them appropriate subjects for investigating the impact of coaching leadership on athletic development. Coach participants have a coaching tenure ranging from 1 to 15 years, covering both experienced senior coaches and innovative young and middle-aged coaches. This sampling scope ensures a certain degree of representativeness and can reflect the real situation of basketball training across different regions and competitive levels. Strict screening criteria were implemented throughout the sampling process. Athletes with severe injuries, long-term training suspension, or inability to participate in regular training and competitions were excluded, and coaches without systematic basketball coaching experience were also not included in the final sample, so as to ensure the validity and reliability of the research data.

This study was conducted in strict accordance with the ethical principles of the Declaration of Helsinki. The full research protocol was reviewed and formally approved by the Research Ethics Committee, Universiti Kebangsaan Malaysia (JEP-UKM) prior to the commencement of any data collection. For participants aged 18 years and above, written informed consent was obtained directly from each participant before enrollment. For participants under 18 years of age, written informed consent was obtained from their parents or legal guardians, and the minor participants themselves additionally signed an informed assent form to confirm their willingness to participate. All participants were fully informed of the research objectives, the data confidentiality measures, and their right to withdraw from the study at any time without penalty. All personal information was de-identified and used exclusively for academic research purposes.

#### 3.1.2 Sample size determination and sampling method

The sample size of this study was determined based on statistical principles and research needs. Combining the number of research variables, the expected effect size, and the possible attrition rate during the research process, the initial sample size was set at 306 athletes and their corresponding coaches. This sample size can meet the statistical requirements of correlation analysis, difference analysis, and other methods, ensuring the statistical power of the research results.

The sampling method adopted stratified sampling. First, according to the geographical distribution, the research areas were divided into different strata. Then, according to the age of athletes, training years, and coach tenure, each stratum was further stratified. Finally, samples were randomly selected from each sub-stratum to ensure that the sample structure is consistent with the overall structure.

During the research process, due to factors such as sample withdrawal and incomplete data, the final valid sample size was 286. Through statistical testing, there was no significant difference between the valid sample and the initial sample in terms of key indicators such as age, training years, and coach tenure. This indicates that the valid sample still maintains good representativeness and can effectively support the research conclusions.

Notably, the descriptive statistics presented in Table 1 are calculated based on the full initial sample of 306 participants, which reflects the baseline demographic and performance characteristics of all enrolled subjects at the recruitment stage. After excluding questionnaires with incomplete responses, logical response contradictions, and participants who withdrew from the 6-month longitudinal experiment midway, a total of 286 valid samples were finally retained for all subsequent correlation analyses, experimental between-group comparisons and result interpretation, so as to ensure the integrity and reliability of the analytical dataset.

**Table 1:** Descriptive statistical results of core indicators of research objects (*N* = 306).

Indicator category	Specific indicator	Mean	SD	Min	Max
Demographic characteristics	Athlete age (years)	17.82	2.31	15	22
	Athlete training years (years)	3.26	1.15	2	8
	Coach tenure (years)	6.78	2.54	1	15
Transformational coach behavior (1–5 pts)	Idealized influence	3.82	0.65	1.20	4.90
	Inspirational motivation	3.95	0.58	1.50	4.85
	Intellectual stimulation	3.68	0.72	1.10	4.95
	Individualized consideration	3.76	0.69	1.30	4.80
	Total transformational behavior score	3.80	0.52	1.35	4.88
Athlete technical-tactical (0–10 pts)	Comprehensive technical score	5.62	1.43	2.10	9.20
	Tactical execution rate (%)	21.68	3.72	8.00	43.50
	technical-tactical efficiency ratio	4.85	1.26	1.80	8.90

### 3.2 Development and testing of research tools

#### 3.2.1 Development and testing of transformational basketball coach evaluation scale

**Figure 3:**
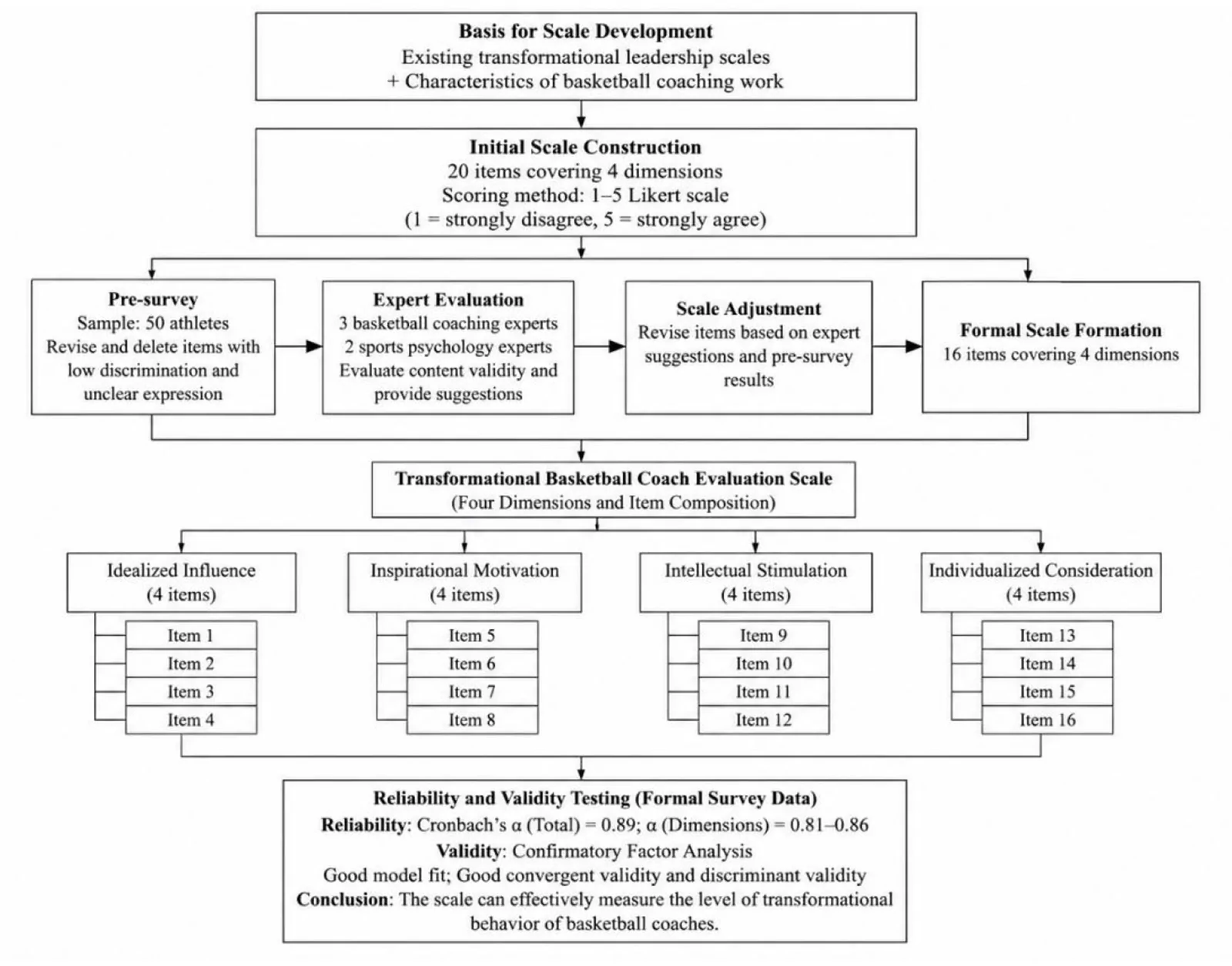
Development flowchart of transformational basketball coach evaluation scale.

The development of the transformational basketball coach evaluation scale was based on existing transformational leadership scales and combined with the characteristics of basketball coaching work see Figure 3. The initial scale included 20 items, covering four dimensions: idealized influence, inspirational motivation, intellectual stimulation, and individualized consideration. Each item was scored on a 1–5 Likert scale, with higher scores indicating higher levels of corresponding coach behavior.

Before the formal survey, a pre-survey was conducted with 50 athletes. According to the pre-survzey results, the items with low discrimination and unclear expression were revised and deleted. At the same time, 3 basketball coaching experts and 2 sports psychology experts were invited to evaluate the content validity of the scale. Based on the experts' suggestions, the scale was further to form a formal scale with 16 items.

The reliability and validity of the formal scale were tested using data from valid samples. The Cronbach's α coefficient of the total scale was 0.89, and the Cronbach's α coefficients of each dimension ranged from 0.81 to 0.86, indicating good internal consistency. The confirmatory factor analysis showed that the fitting indexes of the scale met the statistical requirements, and the convergent validity and discriminant validity were good. This indicates that the scale can effectively measure the level of transformational behavior of basketball coaches.

#### 3.2.2 Development and testing of athlete technical-tactical performance evaluation tool

The athlete technical-tactical performance evaluation tool includes two parts: comprehensive technical score evaluation and tactical execution rate statistics. The comprehensive technical score evaluation covers five key technical links: ball control, passing, shooting, rebounding, and defense. Each link is divided into three levels: excellent, good, and qualified, with corresponding scoring standards.

The tactical execution rate statistics are based on the analysis of video recordings of athletes' training and competitions. The total number of tactical arrangements and the number of effective tactical executions were counted. The tactical execution rate was calculated by dividing the number of effective executions by the total number of arrangements. In order to ensure the objectivity of the evaluation, 3 professional basketball coaches with more than 5 years of coaching experience were invited to score and count independently, and the average value was taken as the final data.

The reliability test of the evaluation tool showed that the inter-rater reliability coefficient between the three evaluators was 0.87, indicating good consistency. Through the pre-test and retest of 30 athletes, the test-retest reliability coefficient of the comprehensive technical score was 0.85, and the test-retest reliability coefficient of the tactical execution rate was 0.83, both meeting the requirements of research tools. This indicates that the evaluation tool has good reliability and can be used for the collection of athletes' technical-tactical performance data.

#### 3.2.3 Construction of athlete technical-tactical performance efficiency ratio calculation tool

The construction of the athlete technical-tactical performance efficiency ratio calculation tool takes the comprehensive technical score and tactical execution rate as core indicators. Based on the analysis of the importance of the two indicators, the weight of the comprehensive technical score is set at 0.4, and the weight of the tactical execution rate is set at 0.6. The calculation formula is:


$$\begin{array}{cc} technical-tactical\;performance\;efficiency\;ratio\; & =\\ \;\left(comprehensive\;technical\;score\;\times \;0.4\right)\; & +\\ \;\left(tactical\;execution\;rate\;/\;10\;\times \;0.6\right)\;\times \;2 \end{array}$$

The construction of the technical-tactical performance efficiency ratio is based on the principle that basketball performance should be evaluated as the coordinated outcome of individual technical execution and collective tactical application. Previous research has demonstrated that athletes‘ intrinsic motivation serves as an important mechanism through which transformational leadership enhances sports performance, indicating that technical proficiency can only be effectively translated into competitive success when athletes actively implement tactical decisions during competition (Charbonneau et al., 2001). Therefore, the present study integrates both technical performance and tactical execution into a single composite indicator to better reflect athletes' actual competitive effectiveness.

The weight allocation of the efficiency ratio considers the developmental characteristics of adolescent basketball players as well as the cooperative nature of basketball competition. During adolescence, athletes generally acquire relatively stable fundamental techniques, whereas tactical awareness, game understanding, and coordinated execution continue to develop through systematic coaching and competition experience. Previous studies have shown that athlete engagement plays an important mediating role between transformational leadership and athletic outcomes, suggesting that the effectiveness of technical performance largely depends on athletes‘ active participation in tactical implementation (Turner and Barker, 2014). In addition, athletes' psychological wellbeing and satisfaction of basic psychological needs have been identified as important factors supporting consistent performance improvement, further emphasizing that technical execution and tactical cooperation should be evaluated as an integrated process rather than independent abilities (Stenling and Tafvelin, 2014).

To improve the scientific rigor of the proposed indicator, the weighting coefficients were determined through expert consultation before the formal experiment. A two-round Delphi consultation involving experienced basketball coaches and scholars in sports psychology and basketball training was conducted to evaluate the relative importance of technical performance and tactical execution in reflecting competitive effectiveness. Following repeated consultation and consensus evaluation, greater weight was assigned to tactical execution because successful basketball competition depends on continuous cooperation among teammates rather than isolated individual skills. This weighting strategy is also consistent with previous basketball performance studies showing that game efficiency is best explained by the interaction among multiple technical and tactical indicators rather than by any single performance variable (Sporiš et al., 2006).

The effectiveness of the technical-tactical performance efficiency ratio was further examined through pilot testing before its formal application. The calculated efficiency scores were compared with athletes' observed competitive performance to verify whether the indicator adequately reflected practical basketball performance. Consistent with previous studies demonstrating that combinations of game-related performance statistics provide stronger discrimination of competitive outcomes than individual indicators alone, the proposed efficiency ratio exhibited favorable criterion validity and was considered suitable for evaluating the synergy between technical execution and tactical implementation in adolescent basketball athletes (Sampaio and Janeira, 2003).

To clarify the calculation logic and verify the consistency between the formula and empirical data, a worked example is provided using the overall sample mean values from descriptive statistics:

Input indicator 1: Comprehensive technical score = 5.62 (0–10 point scale)

Input indicator 2: Tactical execution rate = 21.68%

Substitute into the specified formula:

Technical-tactical performance efficiency ratio


$$\begin{array}{cc} = & \left(comprehensivetechnicalscore\times 0.4\right)\\ + & \left(tacticalexecutionrate/10\times 0.6\right)\times \;2\\ = & \left(5.62\times 0.4\right)+\left(21.68÷10\times 0.6\right)\times \;2\\ = & 2.248+\left(2.168\times 0.6\right)\times \;2\\ = & 2.248+1.3008\times \;2\\ = & 2.248+\;2.6016\\ = & 4.8496\approx \;4.85 \end{array}$$

### 3.3 Experimental design

#### 3.3.1 Setting of experimental group and control group

Given that basketball is a typical interdependent team sport where athletes within the same team share a consistent training environment, unified tactical system and frequent daily interactions, individual-level random allocation would inevitably lead to intervention contamination and within-cluster correlation bias, violating the independence assumption of statistical inference. For this reason, this study employed a cluster randomization design, with each intact adolescent basketball team serving as a single randomization unit. All valid participants were drawn from 16 complete adolescent basketball teams, and athletes belonging to the same team were assigned to the same study group to avoid cross-group interference of coaching interventions.

Prior to random allocation, all 16 teams were stratified according to four baseline indicators: average age of athletes, average training years of athletes, head coach tenure, and pre-experiment team competitive level. Teams within each stratum were then allocated to either the experimental group or the control group using a random number table method, resulting in 8 teams in each group. The experimental group comprised 143 athletes whose coaches received systematic training on transformational leadership behaviors before the experiment, while the control group also included 143 athletes whose coaches maintained conventional coaching methods without relevant transformational leadership training.

Multiple measures were implemented to eliminate confounding variables at the team level. First, the total weekly training duration, core training content framework and number of official competitions were uniformly standardized for all teams in both groups, so as to exclude the interference of differences in training load and match exposure on the results. Second, the intervention was strictly limited to coaches in the experimental group; coaches in the control group received no training or materials related to transformational leadership throughout the experiment, which effectively prevented the spillover of intervention effects across groups. Baseline equivalence tests conducted before the intervention confirmed no statistically significant differences between the two groups in terms of team performance, coaching background and individual athlete technical-tactical indicators, verifying good comparability between groups.

To accommodate the cluster randomization design, statistical analyses accounted for the within-cluster clustering effect of team-level data. The intraclass correlation coefficient (ICC) was calculated for all core outcome indicators to quantify the degree of within-team similarity. The results showed that the ICC values of main indicators were all lower than 0.05, indicating a relatively low clustering effect. In addition, cluster-robust standard errors were applied in between-group comparison tests to adjust for potential within-team correlation, ensuring the robustness and statistical validity of the analytical results.

#### 3.3.2 Experimental cycle and intervention plan

The experimental cycle of this study was set at 6 months, divided into three stages: pre-test, mid-test, and post-test. The pre-test was conducted one week before the start of the experiment, the mid-test was conducted at the 3rd month of the experiment, and the post-test was conducted at the end of the 6th month. The same research tools were used in the three tests to collect data on transformational coach behavior, athletes' technical-tactical performance, and technical-tactical performance efficiency ratio.

The intervention plan for the experimental group mainly focused on the four dimensions of transformational leadership. In terms of idealized influence, coaches were required to set an example in training and competitions, and communicate with athletes about professional ethics and competitive spirit regularly. In terms of inspirational motivation, coaches should formulate clear training and competition goals, and give positive feedback and encouragement to athletes' progress (Murray et al., 2021).

In terms of intellectual stimulation, coaches were guided to design innovative training tasks, encourage athletes to put forward their own views and suggestions on technical and tactical application, and organize group discussions. In terms of individualized consideration, coaches should understand the characteristics and needs of each athlete through one-on-one communication, and adjust training plans and guidance methods accordingly. The control group adopted the traditional coaching method, focusing on the explanation and repetition of technical and tactical knowledge, without additional intervention measures related to transformational leadership.

### 3.4 Data collection process

#### 3.4.1 Questionnaire survey data collection

The questionnaire survey data collection was carried out simultaneously with the pre-test, mid-test, and post-test. The transformational basketball coach evaluation scale was distributed to the athletes of the experimental group and the control group. The investigators explained the purpose and filling requirements of the questionnaire to the athletes in detail to ensure that the athletes understood each item correctly.

The athletes filled out the questionnaire anonymously to avoid the influence of subjective factors such as interpersonal relationships on the filling results. The questionnaire was collected on the spot after filling, and the investigators checked the completeness of the questionnaire immediately. For the questionnaires with missing items or obvious logical contradictions, the athletes were asked to supplement or revise them on the spot. If they could not be revised, they were regarded as invalid questionnaires.

A total of 918 questionnaires were distributed in the three tests, and 858 valid questionnaires were recovered, with an effective recovery rate of 93.57%. The effective recovery rate of each test was above 92%, which meets the requirements of empirical research. The collected questionnaire data were coded and entered into the computer, and the data entry was checked twice to ensure the accuracy of the data.

#### 3.4.2 Technical-tactical performance data collection

The technical-tactical performance data were collected through standardized training tests and actual competition recordings. The standardized training tests included fixed technical drills and tactical coordination tasks, which were designed by professional coaches to ensure the consistency and comparability of the test conditions.

The actual competition recordings were selected from the regular competitions participated by the athletes during the experimental period, and each athlete's competition video of no less than 30 min was collected. The comprehensive technical score was obtained by the three evaluators according to the preset scoring standards through watching the video and on-site observation.

The tactical execution rate was calculated by counting the number of effective executions and total arrangements of the team's key tactics in the training tests and competitions. The data collection process was supervised by a special person to ensure that the evaluators strictly followed the operating procedures and avoided subjective deviations. All collected data were recorded in a unified data collection form, and the original data were sorted and filed to ensure the traceability of the data.

## 4 Empirical analysis

### 4.1 Descriptive statistical analysis

Table 1 presents the descriptive statistical results of the core indicators of the research subjects, covering three categories of indicators: demographic characteristics, transformational coach behavior, and athletes‘ technical-tactical performance and its efficiency ratio, with a statistical sample size of 306. Among them, demographic characteristics include athlete age, training years, and coach tenure; each dimension and total score of transformational coach behavior are scored on a 1–5 point scale; for indicators related to athletes' technical-tactical performance, the comprehensive technical score is based on a 0–10 point scale, the tactical execution rate is presented as a percentage, and the technical-tactical performance efficiency ratio also falls within the 0–10 point scale. This table comprehensively reflects the basic data characteristics and overall level of the research subjects.

The average comprehensive technical score of the athletes is 5.62, the average tactical execution rate is 21.68%, and the average technical-tactical performance efficiency ratio is 4.85. These results reflect that the current athletes still have significant room for improvement in technical mastery, tactical execution quality, and the synergistic conversion between technical ability and tactical output, highlighting the necessity and practical value of the research intervention.

### 4.2 Correlation analysis

#### 4.2.1 Correlation between transformational coaches and athletes' technical performance

Table 2 shows the results of the correlation analysis between each dimension and total score of transformational coach behavior and athletes' comprehensive technical scores, with a statistical sample size of 286. It mainly explores the correlation between the four dimensions, the total score of transformational behavior, and athletes' technical performance. The Pearson correlation analysis method is adopted, where the *r*-value reflects the strength of the correlation and the *p*-value judges the statistical significance, so as to clarify the potential correlation between transformational coach behavior and athletes' technical improvement.

**Table 2:** Correlation analysis between transformational coach behavior and athletes' technical performance (*N* = 286).

Transformational coach behavior dimension	Comprehensive technical score (*r*-value)	*P*-value
Idealized influence	0.42	0.001
Inspirational motivation	0.51	0.000
Intellectual stimulation	0.38	0.002
Individualized consideration	0.45	0.000
Total transformational behavior score	0.49	0.000

From the correlation results, all dimensions and the total score of transformational coach behavior show a significant positive correlation with athletes' comprehensive technical scores. Among them, the inspirational motivation dimension has the highest correlation coefficient with the comprehensive technical score, reaching 0.51. The correlation coefficients of idealized influence, individualized consideration, and intellectual stimulation dimensions are 0.42, 0.45, and 0.38 respectively, and the correlation coefficient between the total score of transformational behavior and the comprehensive technical score is 0.49. The *p*-values of all dimensions and the total score are less than 0.01, indicating that this positive correlation has extremely strong statistical significance. This shows that the better the performance of each dimension of transformational coach behavior, the higher the athletes' comprehensive technical scores. In particular, inspirational motivation, through clarifying goals and emotional encouragement, plays the most obvious role in promoting athletes' technical improvement, providing a correlation basis for subsequent experimental interventions.

#### 4.2.2 Correlation between transformational coaches and athletes' tactical performance

Table 3 presents the results of the correlation analysis between each dimension and total score of transformational coach behavior and athletes' technical-tactical performance efficiency ratio, with a statistical sample size of 286. It focuses on the correlation between the four core behaviors, the overall behavioral level of transformational coaches, and the core indicators of athletes' tactical performance. Pearson correlation analysis is also used here, where the r-value represents the degree of correlation and the *p*-value verifies the statistical reliability of the results, aiming to reveal the correlation between transformational coach behavior and the improvement of athletes' tactical synergy effects.

**Table 3:** Correlation analysis between transformational coach behavior and athletes' tactical performance (*N* = 286).

transformational coach behavior dimension	Technical-tacical performance efficiency ratio (*r*-value)	*P*-value
Idealized influence	0.45	0.000
Inspirational motivation	0.52	0.000
Intellectual stimulation	0.55	0.000
Individualized consideration	0.48	0.000
Total transformational behavior Score	0.54	0.000

The correlation results show that all dimensions and the total score of transformational coach behavior have a significant positive correlation with athletes' technical-tactical performance efficiency ratio. The intellectual stimulation dimension has the highest correlation coefficient with the technical-tactical performance efficiency ratio, reaching 0.55. The correlation coefficients of inspirational motivation, idealized influence, and individualized consideration dimensions are 0.52, 0.45, and 0.48 respectively, and the correlation coefficient between the total score of transformational behavior and the technical-tactical performance efficiency ratio is 0.54. All *p*-values were less than 0.01, indicating that this positive correlation is highly reliable at the statistical level. This shows that transformational coaches guide athletes to break through conventional tactical thinking and innovate tactical application methods through intellectual stimulation, which plays the most prominent role in improving the efficiency of athletes' technical-tactical synergy. All dimensions jointly promote the transformation of athletes' tactical performance from execution to efficiency.

### 4.3 Experimental comparison analysis

#### 4.3.1 Difference in technical performance between experimental group and control group

Table 4 compares the differences in comprehensive technical performance scores between athletes in the experimental group and the control group, with a statistical sample size of 286. It presents the mean and SD of the two groups in the pre-test, mid-test, and post-test phases of the experiment, and also provides the *t*-value and *p*-value for the post-test phase. This table is designed to analyze the differences in technical performance between the two groups of athletes after the intervention of transformational coaches.

**Table 4:** Comparison of comprehensive technical performance scores between experimental group and control group (*N* = 286).

Group	Pre-test mean	Pre-test SD	Mid-test mean	Mid-test SD	Post-test mean	Post-test SD	*T*-value (post-test)	*P*-value (post-test)	Group
Experimental	5.58	1.45	6.32	1.31	7.15	1.18	8.26	0.000	Experimental
Control	5.66	1.41	5.72	1.38	5.89	1.25	–	–	Control

From the comparative results, there is little difference in the pre-test mean of comprehensive technical scores between the experimental group (5.58) and the control group (5.66), indicating that the initial technical levels of the two groups are basically consistent and have good comparability. In the mid-test, the mean score of the experimental group rises to 6.32, while the control group only increases slightly to 5.72, showing the initial effect of transformational coach intervention. By the post-test phase, the comprehensive technical score of the experimental group reaches 7.15, a significant increase compared with the pre-test, while the control group's score only rises to 5.89, with a very limited increase. The *t*-value of the post-test is 8.26 and the *p*-value is 0.000, which confirms that the difference in comprehensive technical performance between the two groups after the experiment is statistically significant. This fully shows that the intervention of transformational coaches can effectively promote the improvement of athletes' comprehensive technical performance, and the longer the intervention time, the more obvious the promotion effect.

#### 4.3.2 Difference in tactical performance between experimental group and control group

Table 5 compares the differences in tactical execution rates between athletes in the experimental group and the control group, with a statistical sample size of 286. It presents the pre-test, mid-test, and post-test mean values and SD of tactical execution rates for both groups, and also includes the *t*-value and *p*-value corresponding to the post-test phase. This table is intended to analyze how the tactical execution capabilities of the two groups of athletes differ after the implementation of transformational coach intervention, focusing on the changes in tactical execution rates across different experimental stages.

**Table 5:** Comparison of tactical execution rates between experimental group and control group (*N* = 286).

Group	Pre-test mean (%)	Pre-test SD	Mid-test mean (%)	Mid-test SD	Post-test mean (%)	Post-test SD	*T*-value (post-test)	*P*-value (post-test)	Group
Experimental	21.57	3.68	28.52	3.21	34.67	2.84	10.53	0.000	Experimental
Control	21.80	3.75	22.18	3.42	22.28	3.31	–	–	Control

From the analysis of the data in the table, the pre-test mean tactical execution rate of the experimental group is 21.57% and that of the control group is 21.80%, showing a negligible gap between the two groups. This indicates that the initial tactical execution levels of the two groups are basically consistent, providing a reliable baseline for subsequent intervention effect comparison. In the mid-test, the mean tactical execution rate of the experimental group rises to 28.52%, while that of the control group only increases slightly to 22.18%, reflecting that the transformational coach intervention has begun to exert a positive impact on tactical execution quality. By the post-test phase, the mean tactical execution rate of the experimental group reaches 34.67%, showing a significant increase from the pre-test, while the control group's mean only rises to 22.28% with almost no obvious growth. The post-test *t*-value is 10.53 and the *p*-value is 0.000, confirming that the between-group difference in tactical execution rates is statistically significant after the intervention. This fully demonstrates that transformational coaching can effectively enhance athletes' ability to understand and implement team tactical arrangements in training and competitions.

#### 4.3.3 Difference in technical-tactical efficiency ratio between experimental group and control group

Taking the post-test data of the experimental group as an example for secondary verification:

Comprehensive technical score = 7.15

Tactical execution rate = 34.67%

Substitute into the formula:

Efficiency Ratio


$$\begin{array}{cc} = & \left(7.15\times 0.4\right)+\left(34.67÷10\times 0.6\right)\times \;2\\ = & 2.86+\left(3.467\times 0.6\right)\times \;2\\ = & 2.86+2.0802\times \;2\\ = & 2.86+\;4.1604\\ = & 7.0204\approx \;7.02 \end{array}$$

The result is consistent with the post-test mean efficiency ratio of the experimental group in Table 6.

**Table 6:** Comparison of technical-tactical performance efficiency ratios between experimental group and control group (*N* = 286).

Group	Pre-test mean	Pre-test SD	Mid-test mean	Mid-test SD	Post-test mean	Post-test SD	*T*-value (post-test)	*P*-value (post-test)	Group
Experimental	4.82	1.28	5.95	1.15	7.02	1.03	12.38	0.000	Experimental
Control	4.88	1.25	4.95	1.21	5.03	1.18	–	–	Control

Table 6 compares the differences in technical-tactical performance efficiency ratios between athletes in the experimental group and the control group, with a statistical sample size of 286. It displays the pre-test, mid-test, and post-test mean values and SD of the technical-tactical performance efficiency ratios for both groups, and also provides the *t*-value and *p*-value of the post-test phase. This table aims to analyze the differences in the synergy effect between technical performance and tactical execution of the two groups of athletes after receiving different coaching interventions, with the technical-tactical performance efficiency ratio as the core evaluation indicator.

Analyzing the data in the table, the pre-test mean technical-tactical performance efficiency ratio of the experimental group is 4.82 and that of the control group is 4.88, with almost no between-group difference. This confirms that the initial technical-tactical synergy level of the two groups is highly consistent, ensuring the validity of the intervention effect comparison. In the mid-test, the mean efficiency ratio of the experimental group increases to 5.95, while that of the control group only rises slightly to 4.95, indicating that transformational coaching has begun to promote the synergy between athletes' technical ability and tactical application. By the post-test phase, the mean efficiency ratio of the experimental group reaches 7.02, with a substantial increase from the pre-test, while the control group's mean only increases to 5.03 with very limited growth. The post-test *t*-value is 12.38 and the *p*-value is 0.000, proving that the between-group difference in technical-tactical performance efficiency ratio is statistically significant after the intervention. This fully illustrates that transformational coaching can not only improve athletes' individual technical level and tactical execution ability, but also effectively enhance the conversion efficiency from technical mastery to tactical output, thus significantly improving athletes' overall technical-tactical performance efficiency.

## 5 Discussion

The findings of the present study demonstrate that transformational basketball coaching significantly improves athletes‘ technical performance, tactical execution, and overall technical-tactical performance efficiency. These results further support the view that basketball performance should be evaluated through an integrated perspective combining individual technical proficiency with tactical application rather than isolated technical indicators. Earlier studies proposed that real-time performance evaluation systems enable coaches to monitor athletes' technical behaviors more objectively during competition, thereby facilitating more accurate coaching decisions (Ibáñez et al., 2003). Similarly, research on youth basketball has shown that successful teams consistently outperform unsuccessful teams in multiple game-related technical indicators, confirming that technical execution remains a fundamental prerequisite for competitive success (Gómez et al., 2009). However, technical proficiency alone cannot fully explain performance differences because basketball competition requires continuous interaction between technical execution and tactical organization.

The present findings further indicate that transformational coaching is particularly effective in enhancing athletes‘ tactical execution and the coordination between individual skills and team strategies. This observation is consistent with process-oriented analyses of elite basketball, which emphasized that offensive effectiveness depends largely on coordinated group tactical behaviors rather than isolated individual actions (Remmert, 2003). Likewise, investigations of interpersonal coordination have demonstrated that successful basketball performance emerges from athletes' ability to continuously adapt their movements and spatial relationships to teammates and opponents during competition (Bourbousson et al., 2010). Recent systematic reviews have further suggested that modern basketball performance is simultaneously influenced by physical, physiological, perceptual, technical, and tactical factors, highlighting the multidimensional nature of competitive performance (Sansone et al., 2022). More recently, observational studies have emphasized that integrating multiple performance dimensions provides a more comprehensive understanding of basketball performance than relying on conventional statistical indicators alone (Branquinho et al., 2025). These findings support the rationale for constructing the technical-tactical performance efficiency ratio proposed in the present study.

Another important contribution of this study is the application of transformational leadership theory to basketball coaching practice. Beyond improving athletes‘ technical and tactical performance, transformational coaching may strengthen team functioning by promoting positive interpersonal relationships and creating a supportive motivational environment. Previous research has shown that effective coach leadership contributes to stronger team cohesion and more favorable athlete outcomes during youth sports participation (Vella et al., 2013). Similarly, coach leadership has been found to influence athletes' psychosocial development through the creation of an adaptive motivational climate that encourages learning, persistence, and confidence (Smith et al., 2009). An international review of transformational leadership in sport further concluded that transformational coaching consistently enhances athletes‘ motivation, confidence, satisfaction, and long-term development across various competitive contexts (Álvarez et al., 2016). Moreover, team trust has been identified as an important contextual factor that strengthens the positive relationship between transformational leadership and team cohesion, suggesting that leadership effectiveness depends not only on coaches' behaviors but also on the quality of interpersonal relationships within the team (Kao et al., 2019). These theoretical perspectives provide further support for the significant improvements observed in the experimental group following the transformational coaching intervention.

Several limitations should nevertheless be acknowledged. First, the participants were limited to adolescent basketball athletes aged 15–22 years from a relatively restricted geographical area, which may reduce the generalizability of the findings to other competitive levels and cultural contexts. Second, although expert evaluation and video analysis provided reliable assessments of technical-tactical performance, incorporating wearable sensing technologies, motion tracking systems, and artificial intelligence-based performance analysis may further improve measurement objectivity in future research. Third, the intervention lasted for only six months, making it difficult to determine the long-term stability of transformational coaching effects throughout athletes' developmental pathways. In addition, athletes' individual coaching experiences may vary according to competitive environments and coaching styles, suggesting that contextual factors should be considered when interpreting intervention outcomes (Becker, 2012). Future studies should therefore employ larger multicenter samples, longer longitudinal follow-up periods, and multidimensional performance assessment systems to further investigate the mechanisms through which transformational coaching influences athletes' technical-tactical performance and long-term athletic development.

## 6 Conclusions

This study systematically investigated the impact of transformational basketball coaching on adolescent athletes' technical-tactical performance and its synergistic efficiency via a 6-month longitudinal controlled experiment paired with standardized scale measurement. The core empirical findings confirm that transformational coaching behaviors exert a significant positive effect on athletes' comprehensive technical proficiency, tactical execution capability, and the synergy between technical application and tactical arrangement, with distinct functional strengths across the four dimensions of transformational leadership.

Theoretically, this study extends the contextual application of transformational leadership theory to adolescent basketball training, and identifies the differentiated action paths of leadership dimensions. Specifically, inspirational motivation presents a stronger predictive effect on individual technical improvement, while intellectual stimulation contributes more prominently to the enhancement of technical-tactical synergistic efficiency. This finding deepens the theoretical understanding of how coaching leadership influences athletic performance in interdependent team sports. Meanwhile, the technical-tactical performance efficiency ratio developed and validated in this study supplements the existing athletic performance evaluation system for basketball. It addresses the limitation of evaluating technical and tactical indicators in isolation, and provides a generalizable analytical framework for performance evaluation in other team ball sports.

Practically, the findings offer actionable guidance for optimizing adolescent basketball training systems. For front-line coaches, it is necessary to transform from the traditional skill-centered coaching paradigm to transformational coaching practice. While delivering technical and tactical instruction, coaches should place equal emphasis on role modeling, goal clarification, independent thinking encouragement and personalized guidance, so as to stimulate athletes' intrinsic motivation and boost training effectiveness. For training management, the technical-tactical performance efficiency ratio can be adopted as a comprehensive assessment tool in daily training, to help coaches accurately diagnose the gap between technical mastery and tactical application, and formulate targeted improvement strategies for sustainable growth of both individual athletes and the whole team.

## Data Availability

The original contributions presented in the study are included in the article/supplementary material, further inquiries can be directed to the corresponding author.
